# A punctuated equilibrium analysis of the climate evolution of cenozoic exhibits a hierarchy of abrupt transitions

**DOI:** 10.1038/s41598-023-38454-6

**Published:** 2023-07-12

**Authors:** Denis-Didier Rousseau, Witold Bagniewski, Valerio Lucarini

**Affiliations:** 1 Géosciences Montpellier, Université Montpellier, Montpellier, France; 2 Institute of Physics-CSE, Division of Geochronology and Environmental Isotopes, Silesian University of Technology, Gliwice, Poland; 3 Lamont Doherty Earth Observatory, Columbia University, Palisades, NY USA; 4 Ecole Normale Supérieure—Paris Sciences et Lettres, Laboratoire de Météorologie Dynamique, Paris, France; 5 Department of Mathematics and Statistics, University of Reading, Reading, UK; 6 Centre for the Mathematics of Planet Earth, University of Reading, Reading, UK; 7 School of Systems Science, Beijing Normal University, Beijing, People’s Republic of China

**Keywords:** Climate sciences, Palaeoclimate

## Abstract

The Earth’s climate has experienced numerous critical transitions during its history, which have often been accompanied by massive and rapid changes in the biosphere. Such transitions are evidenced in various proxy records covering different timescales. The goal is then to identify, date, characterize, and rank past critical transitions in terms of importance, thus possibly yielding a more thorough perspective on climatic history. To illustrate such an approach, which is inspired by the punctuated equilibrium perspective on the theory of evolution, we have analyzed 2 key high-resolution datasets: the CENOGRID marine compilation (past 66 Myr), and North Atlantic U1308 record (past 3.3 Myr). By combining recurrence analysis of the individual time series with a multivariate representation of the system based on the theory of the quasi-potential, we identify the key abrupt transitions associated with major regime changes that separate various clusters of climate variability. This allows interpreting the time-evolution of the system as a trajectory taking place in a dynamical landscape, whose multiscale features describe a hierarchy of metastable states and associated tipping points.

## Introduction

Early evidence of abrupt transitions in Camp Century and Dye 3 Greenland ice cores^1,2^ attracted a lot of attention from the paleoclimatic community before being well acknowledged and understood. These findings gave evidence of a sequence of previously unknown abrupt climatic variations. However, such transitions did not seem to agree with other marine and terrestrial records, which led to considerable debate in the field^3–5^. Over the course of several decades spent retrieving and studying more detailed paleorecords, the existence of these rapid climatic variations, known as Dansgaard-Oeschger events (DO), has become well accepted. Further support for these findings has recently been provided by the identification of additional abrupt transitions from the NGRIP ice core, which has been made possible by the increased temporal resolution of the record^6^. These additional events correspond to changes of either short duration or amplitude in the stable ^18^O over ^16^O isotopes ratio δ^18^O that visual or standard statistical inspections do not necessarily flag. We remind that the fractionation of oxygen isotopes can be used to reconstruct local temperature conditions.

The Earth climate has experienced numerous abrupt and critical transitions during its long history, well beyond the specific examples above^7,8^. As discussed in Rothman^9^ following Newell ^10^, rapid variations change are more likely to lead to catastrophic consequences in the biosphere—as in the extreme case of mass extinction events—because it is hard for the evolutionary process to keep pace with shifting environmental conditions. Such transitions are often referred to as climatic tipping points (TPs), associated with possibly irreversible changes in the state of the system. The term TP was originally introduced in social sciences^11^ and made popular more recently by Gladwell^12^. The study of TPs has recently gained broad interest and perspective in Earth and Environmental sciences, especially with regard to the future of our societies under the present climate warming scenarios^13–16^. The term Tipping Elements (TE) was introduced in^15,17^, and subsequently adopted by other authors^14,18^ to characterize the specific components of the Earth System that are likely to experience a TP in the near future as a result of the ongoing climate crisis^16,19–21^. Recently, the concept of TP has been used to define, in turn, rapid societal changes that might lead to potentially positive impacts in terms of addressing the climate crisis^22,23^.

Here, we want to investigate critical transitions in the Earth climate history by looking at two key high-resolution datasets that show evidence of abrupt transitions. The first dataset is the CENOGRID benthic δ^18^O and δ^13^C record corresponding to the compilation of 14 marine records over the past 66 Myr, from the Cretaceous-Paleogene (K–Pg) extinction event till present^24^. The second dataset comprises the North Atlantic U1308 benthic δ^18^O, δ^13^C and δ^18^O bulk carbonate time series covering the past 3.3 Myr^25^.

While visual evidences of abrupt transitions have already been discussed for these datasets, we wish to identify key abrupt thresholds by applying the recurrence quantification analysis (RQA) to each individual univariate time series and supplementing it with the Kolmogorov–Smirnov (KS) test^26^, see^27^ and discussion below. Then, the selected transitions are discussed in the context of the Earth climate history allowing the definition of dynamical succession of abrupt transitions. Such transitions are then interpreted taking into account the evolution of key climate factors such as CO_2_ concentration, average global sea level, and depth of the carbonate compensation.

The existence of TPs is intimately related to the multistability properties of the climate system, which have long been recognised in different contexts; see e.g.^28–31^ and discussion in^32,33^. The multistability of the climate system comes the presence of more than one possible climate states for a given set of boundary conditions^34^. While earlier analyses have mostly evidenced the possibility of bistable behaviour, multistability can indeed include multiple competing states^35–38^. Recently, it has been proposed that the metastability properties of the climate system can be understood by interpreting the climate evolution as a diffusion process taking place in an effective dynamical landscape^37,39,40^defined by the the Graham’s quasi-potential^41^. The local minima of the quasi-potential indicate the competing metastable states, with the transitions between such states occurring preferentially through the saddles of the quasi-potential. Such a viewpoint mirrors earlier proposals for interpreting biological evolution, namely the Waddington’s epigenetic landscape^42–46^, see also relevant literature associated with synthetic evolution models like Tangled Nature^49,50^, and foresees the climatic transitions associated with the TPs as a manifestation of a dynamics characterized by punctuated equilibria^47–50^, The interplay between periods of stasis and rare transitions between competing metastable states seem to reflect the fact that climate fluctuations behave according to the dominance of stabilizing vs destabilizing feedbacks when considering short vs long time scales, respectively^51^. We will see that in most cases the RQA applied to the CENOGRID δ^18^O dataset flags TPs that disagree in terms of dating from those derived from the δ^13^C dataset. This is unsurprising because the two proxy data are sensitive to vastly different climatic processes. Nonetheless, the candidate TPs from both datasets come hand in hand with saddles of the bidimensional quasi-potential estimated from the bivariate time series. This indicates consistency between separate ways of detecting TPs. Additionally, TPs featuring faster characteristic time scales are associated with smaller-scale decoration of the quasi-potential, in agreement with what conjectured in^37^. The analysis of the benthic δ^18^O and δ^13^C suggests that the evolution of the climate in Cenozoic is characterized by a hierarchy of TPs due to an underlying multiscale quasi-potential.

## Results

### Detecting critical transitions of the past 66 Myr—3 Myr history of the earth climate

**Figure 1:**
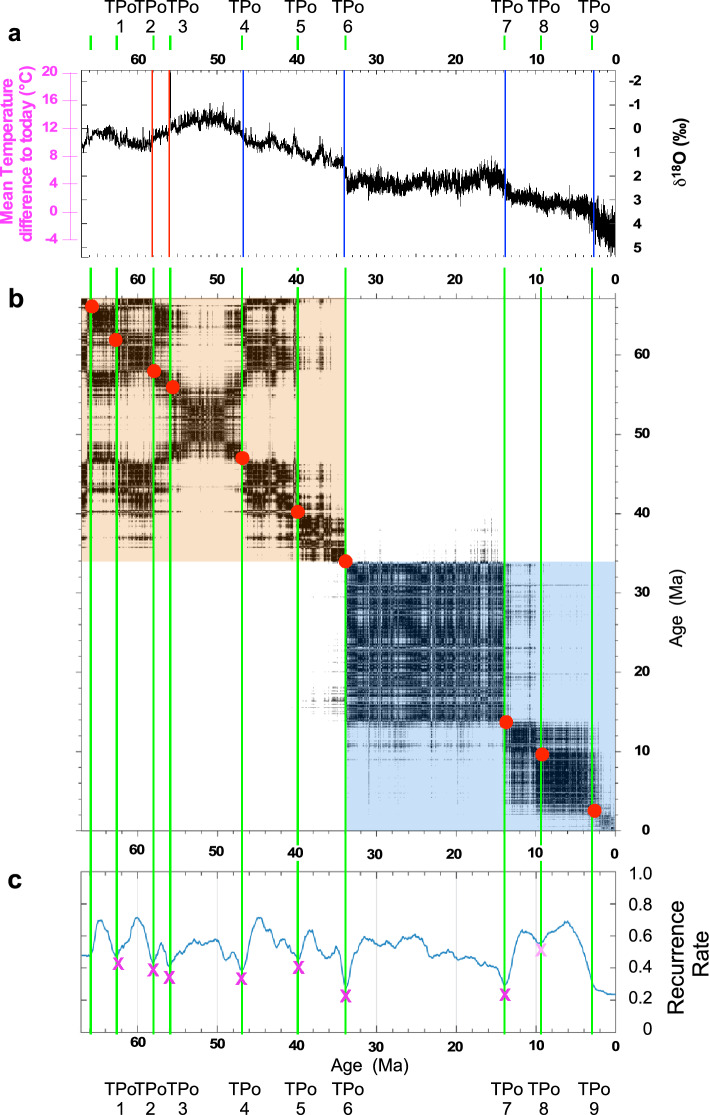
KS test and Recurrence Quantification Analysis (RQA) of CENOGRID benthic δ^18^O. (**a**) Time series in Ma BP with difference of the reconstructed and present Mean Global Temperature in pink). KS test identifying abrupt transitions towards warmer conditions in red and cooler or colder conditions in blue; (**b**) Recurrence plot (RP) with identification of the main two clusters prior and after 34 Ma. The main abrupt transitions identified are highlighted by red circles, and (**c**) Recurrence rate (RR). The pink crosses and vertical green lines indicate the abrupt transitions (TP) detected by the RQA. CENOGRID benthic δ^18^O data are from Westerhold et al. ^24^.

The augmented KS test of the benthic δ^18^O record of the past 66 Ma identifies six major abrupt transitions corresponding to two major warming events at about 58 Ma and 56 Ma, followed by four major coolings at 47 Ma, 34 Ma, 14 Ma and 2.8 Ma respectively (Fig. 1A). The competing metastable states associated with these transitions feature rather long temporal persistence (a long time-window of 1–4 Ma is used, see Suppl. Mat.). These events are classical ones described from the literature^52^, where the first two transitions led to warmer conditions, while the latter four led to colder conditions. The same transitions are identified by employing the recurrence plot (RP) and recurrence rate (RR) analyses^27,53^, which also identify three more events, occurring at around 63 Ma, 40 Ma and 9.7 Ma, see Fig. 1C, Suppl. Tab. 1. We have chronologically labeled these TP_O_1 to TP_O_9, where the lower index refers to the used proxy data. As shown in Fig. 1B, TP_O_6 separates the climate variability in two separate macroclusters prior and after 34 Ma, the well-known Eocene–Oligocene Transition (EOT)^54^, which is a key step in the Cenozoic climate history and is associated with a major extinction event^55,56^.

The older macrocluster corresponds to very warm climate conditions and, using Marwan et al.’s nomenclature^53^, a disrupted variability. The average global temperature was estimated to be between 8 °C and 16 °C above the present day one, with no apparent presence of any major continental ice bodies^24^ (Fig. 1A). Five major transitions are found within this period. They include the 2 major abrupt warmings at 58 Ma (TP_O_2) and 56 Ma (TP_O_3). These two transitions are thresholds towards much warmer oceanic deep water characterizing the first late Paleocene-Eocene hyperthermal^57,58^ and Paleocene-Eocene Thermal Maximum (PETM) ^59,60^ respectively. The third one at about 47 Ma (TP_O_3) represents a transition towards cooler deep waters and named the Early-Middle Eocene cooling^58^. The last one at about 40 Ma (TP_O_4) inaugurates the period of continuous cooling due to the decrease of CO_2_ concentrations that eventually leads to the TP_O_6 event^61^, which is associated with the build-up of the Antarctica ice sheets. TP_O_6 separates two fundamentally different modes of operation of the climate system, corresponding, as discussed later in Sect. 4, to two separate minima of the quasi—potential. From 34 Ma to present day the records feature more positive values of benthic δ^18^O associated with prevailing colder climate conditions. After TP_O_6, the climate featured mostly stationary conditions with a slight warming until the Middle Miocene Climate Transition (TP_O_7) that occurred around 14 Ma^62,63^. The last major transition (TP_O_9) occurred around 2.8 Ma, leading to the Pleistocene and the onset of glaciations in the Northern Hemisphere.

What discussed above provides a more systematic and robust counterpart of the analysis of TPs presented in^24^. In general, we prioritize the information coming from the δ^18^O record because of its very strong link to the Earth’s temperature. Nonetheless, the analysis of the Benthic δ^13^C record performed along the same lines as above provides separate pieces of information on the critical transitions of the Earth’s Climate. Benthic δ^13^C values characterize deep-water ventilation with high δ^13^C values in regions close to deep-water formation area. The KS analysis performed over a time window of 1–4 Myr individuates 14 TPs, with the RP suggesting an additional one, located at around 34 Ma and associated with the EOT. We refer to these 15 TPs associated with the δ^13^C record as TP_C_ 1 to 15; see Suppl. Figure 1, Suppl. Tab. 1. The interval 56.15 Ma–7.15 Ma is well characterized, showing some subclusters distributed around 34 Ma. The 56.15 Ma date groups δ^13^C values above 1‰, at the base of the record, while 7.15 Ma gathers the negative δ^13^C values which mainly occurs at the top of the record. The two periods are characterized by very different climatic conditions. The earlier climate regime features more input of carbon in the ocean while the later climate, instead, is characterized by higher presence of carbon in the atmosphere.

### Discussion of the detected critical transitions

**Figure 2:**
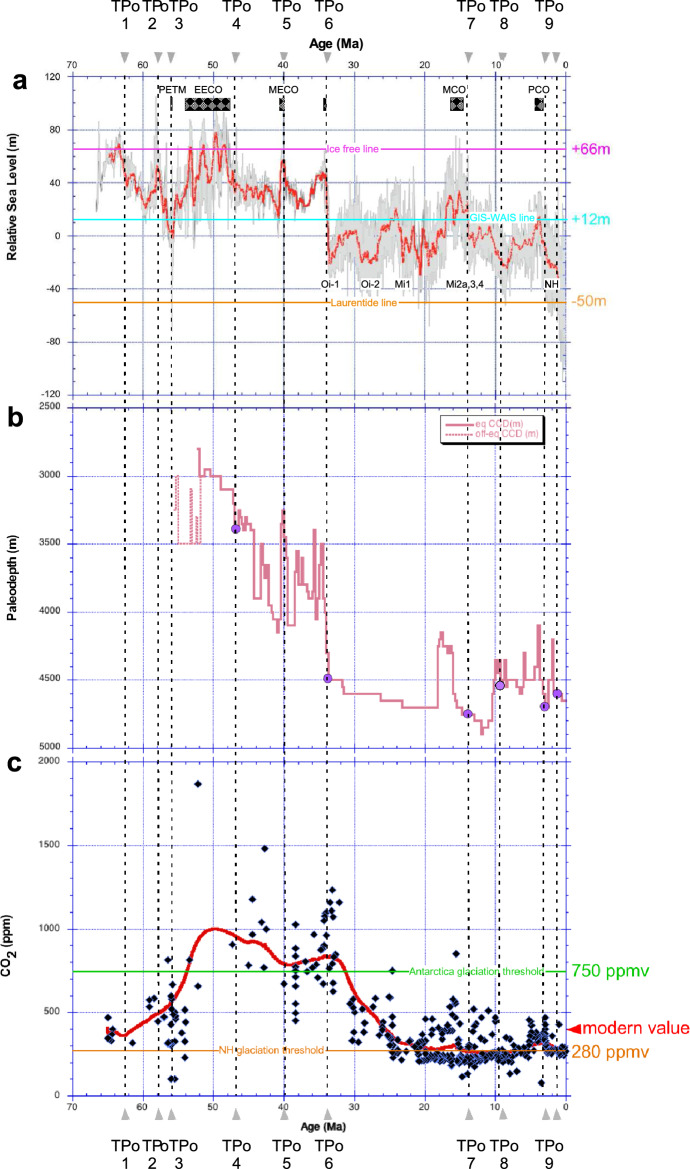
Variation through time of three main climate factors and comparison with the identified abrupt transitions (TP_O_) in the CENOGRID benthic δ^18^O. (**a**) Global Mean Sea Level in meters from Miller et al. ^58^. Identification of particular warm and of glaciation events. The Laurentide, GIW-WAIS and Ice free lines are from Miller et al. ^58^; (**b**) Carbonate Compensation Depth (CCD) in meters from Pälike et al. ^64^. The purple circles identify the TPs on this record; (**c**) Estimate of the CO_2_ concentration in parts per million volume (ppmv) from Beerling & Royer^65^. The Antarctica glaciation threshold at 750 ppmv and the NH glaciation threshold at 280 ppmv lines respectively are from DeConto et al. ^66^.

We next want to analyze TP_O_ 1–9 in relation to different reconstructed paleoclimatic data, namely the global mean sea level (GMSL), the Pacific carbonate compensation depth (CCD), and CO_2_ concentration (Fig. 2). Using benthic foram δ^18^O and Mg/Ca records from high-resolution Pacific cores which were not included in the CENOGRID compilation, Miller et al. ^58^ have reconstructed variations in the GMSL over the past 66 Ma. By measuring the carbonate content in Pacific sediment cores and applying transfer functions, Pälike et al.^64^ have generated a detailed Cenozoic record of the Pacific CCD, which denotes the depth below which carbonates dissolve. Finally, by compiling estimates from various proxies including foram δ^13^C, boron isotopes, stomata, paleosols, Beerling & Royer^65^ have produced a comprehensive Cenozoic record of the CO_2_ concentration. The signature of the TP_O_6 is evident in the three records, corresponding to an abrupt decrease of the GMSL by about 70 m and of the CCD by around 1000 m. Additionally, TP_O_6 marks the start of a progressive decrease in CO_2_ levels, from approximately 750 ppm to values of the order of 280 ppm.

Along the lines of Westerhold et al.^24^, one can identify four competing states,”Warmhouse” (66 Ma-TP_O_1 and TP_O_3- TP_O_5) and ”Hothouse” (TP_O_1-TP_O_3) climates in the earlier, warmer period, followed by the ”Coolhouse” (TP_O_5–TP_O_6) and ”Icehouse” climates (TP_O_6 to present); see Fig. 1. The first two states alternated in a warm-hot-warm sequence under extremely high CO_2_ concentrations^65^ as compared to those measured over the past 800 Kyr in the Antarctic ice cores (Fig. 2C). Before 34 Ma, one finds substantially larger values for GMSL, CCD depth, and CO_2_ concentration whose average values are: + 38 ± 15 m, 4600 ± 150 m, and 630 ± 300 ppmv respectively (Suppl. Tab. 2). Note that the CO_2_ concentration is much higher than the levels observed over the past 800 kyr in the Antarctic ice cores, which represent the reference states for the potential scenarios of climate warming outlined by the IPCC^67^. Conversely, from 34 Ma until present, the Earth experienced much lower CO_2_ concentrations and GMSL, thus generating the classical climate trend towards the recent ice-age conditions^8,24,52^ (Fig. 2A,C). Indeed, the last 34 Myr show average values in GMSL, CO_2_ concentration, and CCD depth of − 3.5 ± 13 m, 330 ± 160 ppmv, and 3500 ± 400 m, respectively (Suppl. Tab. 2), which are much lower than in the older interval. This second set of means has underestimated values since it is based on data from the Miller et al. ^58^ dataset, which ends at 0.9 Ma,

These GMSL, CCD and CO_2_ reconstructions show key transitions that fit with the CENOGRID thresholds deduced from the KS and RR analysis of the benthic δ^18^O as they signal an increase or a decrease in the global mean sea level corresponding roughly to warming or cooling episodes of the Earth history or strong variations in the concentration of atmospheric CO_2_. The variations observed in CO_2_, CCD and GMSL at the 9 identified TPs from the benthic δ^18^O record indicate heterogeneous characteristics prior to the TP6 major threshold (Suppl. Tab. 3). On the contrary, more homogenous features are noticed in the three climate proxies after TP_O_6, translating the occurrence of major reorganizations in the climate system, which become interesting to test at shorter timescales; see discussion below.

## Quasi-potential landscape and critical transitions

**Figure 3:**
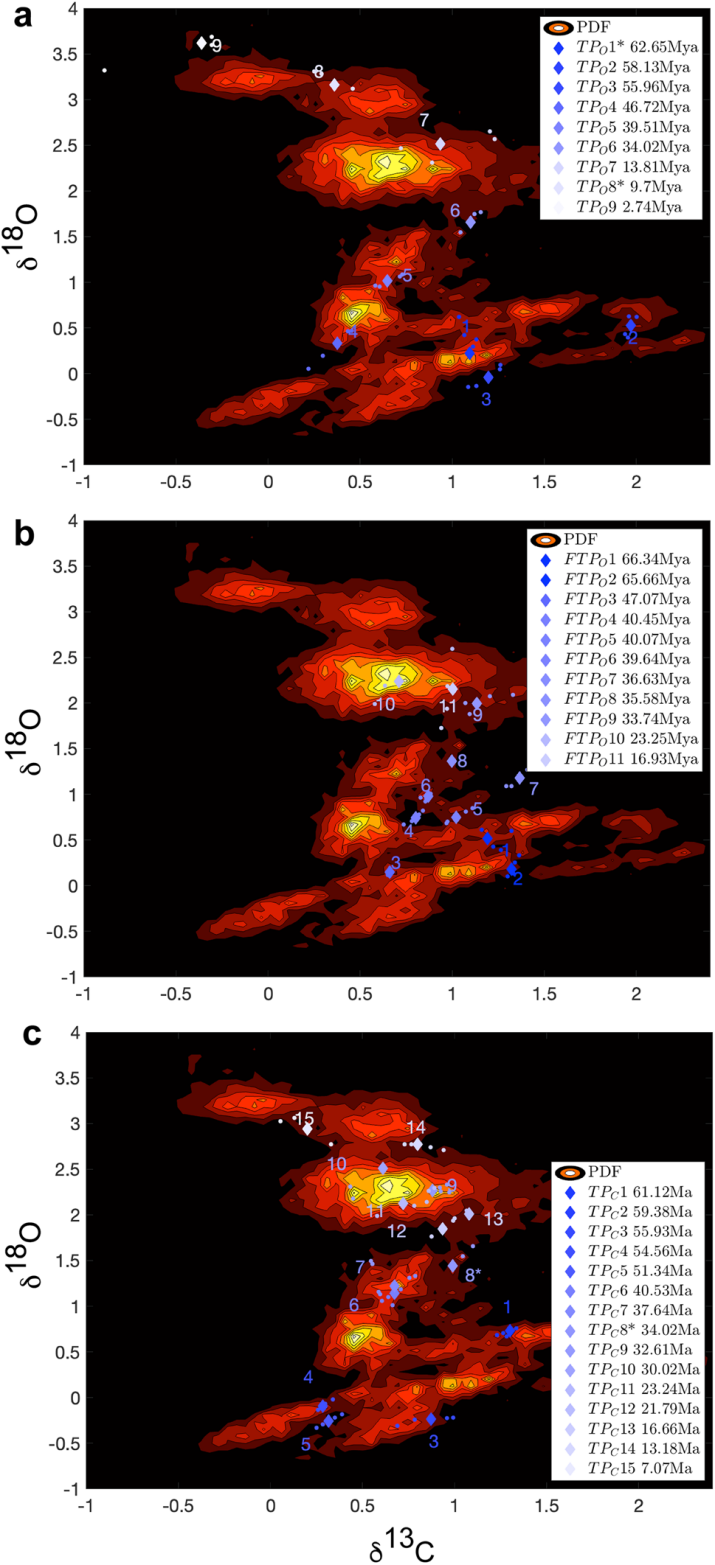
Probability density of the climate system in the projected CENOGRID benthic δ^18^O and δ^13^C. space. (**a**) Chronologically ordered TP_O_s (diamonds) selected according to the KS methodology for δ^18^O with time window 1–4 My are shown. The two extra TP_O_s found via RP are indicated with a *. (**b**) Chronologically ordered fast TP_O_s (FTP_O_s, diamonds) selected according to the KS methodology for δ^18^O with time window 0.25–1 Myr are shown. (**c**) Same as a), but for the δ^13^C record. The approximate timing of the TPs is indicated (rounded to .01 My). The 5 Ky-long portions of trajectories before and after each TP are also plotted.

As discussed in detail in the Methods section, taking inspiration from the use of the Waddington epigenetic landscape to describe evolution^42–46^ and from the theory of punctuated equilibrium^49,50^, it has been proposed in^37,39,40^ to study the global stability properties of the climate system by introducing an effective quasi-potential^41^, which generalizes the classical energy landscape framework often used to study metastable stochastic system. The quasi-potential formalism allows one to study general non-equilibrium system and to associate local maxima of the probability distribution function (pdf) of the system stable states. Additionally, saddles of the pdf are associated with Melancholia (M) states^34^, which are unstable states living in the boundary between different basins of attraction. In the weak-noise limit, noise-induced transitions between competing stable states are expected to go through such M states. What we have done here is to construct the empirical bivariate pdf of climate system in the projected (δ^13^C, δ^18^O) and check whether TP_O_ 1–9 indicated in Fig. 1 correspond, at least approximately, to saddles of the pdf. We find— see Fig. 3A—that, indeed, this is the case for TP_O_s 1, 2, 4, 5, 6, 8, 9, whereas no agreement is found for TP_O_s 3 and 7, which seem to take place in regions where the density is very small and very large, respectively. A very similar version of the bivariate pdf shown here had already been used in Westerhold et al. ^24^ to identify the Icehouse, the Coolhouse, the Warmhouse, and the Hothouse states, as groupings of nearby peaks corresponding to qualitatively similar climates.

A major improvement we propose here is to identify the transitions between such states, as well as less obvious transitions, by looking at the saddle points. TPo6 clearly emerges as the most important transitions, as it basically breaks the pdf into two separate parts. Note that the transitions shown in Fig. 1 are associated, because of the choice of a long time-window, with events that occur over long periods and that lead to persistent changes in the state of the system.

By combining the recurrence analysis with the quasi-potential formalism we can extract further relevant information. It is natural to ask ourselves what happens if, instead, we consider a catalogue of transitions for the δ^18^O record that are detected by considering shorter time windows (0.25 Ma–1 Ma) in the KS procedure. One finds— see Fig. 3B—11 of such transitions (see Suppl. Mat.). Once we report such fast TP_O_s (FTP_O_s) into the empirical bivariate pdf of the climate system in the projected (δ^13^C, δ^18^O) space, we find that they correspond to finer and smaller structures of the pdf as opposed to the case of the TPs. Hence, events that are associated with faster time scales are associated with smaller jumps between secondary maxima in the pdf belong to a hierarchically lower rung than those occurring over longer time scales. This seems to support the proposal made in^37^.

As additional step, we repeat the same analysis leading to Fig. 3A by using, instead, the δ^13^C record, which features, as mentioned above, a total of 15 TP_C_s. Figure 3C shows clearly that the TP_C_s are, for the most part, saddles that had not been flagged by the TP_O_s. We have evidence that the same saddle is crossed more than once in a back-and-forth fashion few millions of years apart (e.g. TP_C_4 & TP_C_5; TP_C_6 & TP_C_7; TP_C_12 & TP_C_13), which supports the dynamical interpretation discussed here. Comparing Fig. 3A and C, one discovers that the same saddle is responsible for TP_O_8 and for TP_C_15 even if the dating is different. Similarly, the same saddle is responsible for TP_O_5 and TP_C_6 & TP_C_7. Two key climatic features that appear as TPs in both records: the PETM is captured by TP_O_3 and TP_C_3 in both records, while TP_O_6 and TP_C_8 represent the EOT.

## The recent past

**Figure 4:**
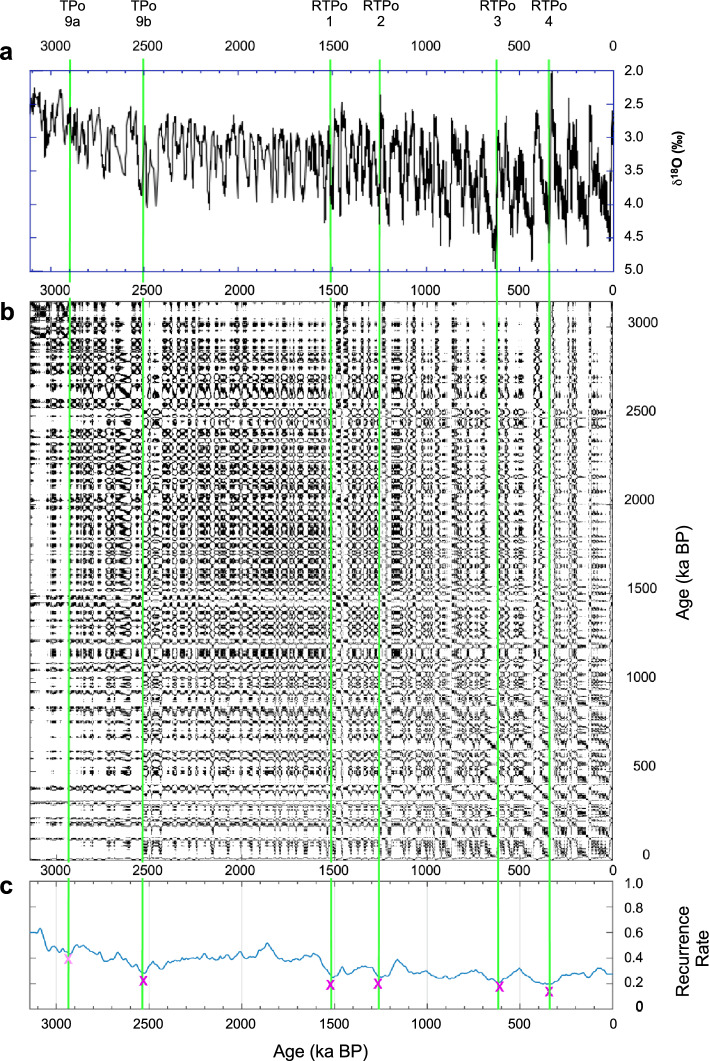
RQA of U1308 benthic δ^18^O. (**a**) Time series in ka BP; (**b**) RP; and (**c**) RR. Pink crosses and green lines as in Fig. 1. TPo9-10 and RTPo1-4 abrupt transitions are identified from the RR. U1308 benthic δ^18^O data are from Hodell & Channell^25^.

The past 3.3 Myr record from North Atlantic core U1308 can be considered as a blow-up of the CENOGRID dataset (Fig. 4). As previously mentioned, the last 3.3 Myr have been defined as an Icehouse climate state, with the appearance, development, and variations of the NHIS^24^, whilst the Antarctic ice sheets had already mostly reached their maximal expansion. The variations in the deep-water temperature, as expressed by the benthic δ^18^O, are interpreted as an indicator of the continental ice volume with clear interglacial-glacial successions^68–70^. The Icehouse state is characterized by a change of the interplay between benthic ${\updelta }$ ^13^C and ${\updelta }$ ^18^O, which corresponds to a new relationship between the carbon cycle and climate^71^. Indeed, one finds a very strong correlation between the two records (Pearson’s coefficient being approximately -0.6). The correlation mainly results from the fact that the time series have approximately a common quasi-periodic behavior due to amplified response to the astronomical forcing as dictated by the Milankovich theory. Note that that the presence of such almost regular resonant oscillations makes the use of the quasi-potential framework not particularly useful for describing the dynamics of the system, so that we will not pursue this approach for the analysis of the Quaternary records.

The KS augmented test and the RQ of the benthic δ^18^O agree in identifying six abrupt transitions dated at approximately 2.93 Ma, at 2.52 Ma, at 1.51 Ma, at 1.25 Ma, at 0.61 Ma and at 0.35 Ma (Fig. 4, Suppl. Tab. 1). They characterize the dynamics of North Hemisphere ice sheets (elevation and spatial expansion) and agree with the classical transitions characterizing the Marine Isotope Stages (MIS) as already observed in numerous records covering the same interval (see Rousseau et al.^72^ and references. therein).

The first two transitions broadly match the previously discussed CENOGRID’s TP_O_9 associated with the onset of the Pleistocene (note that the interval between the two is much smaller than the resolution needed to separate to TP_O_s) and are followed by four more recent TPs associated with the benthic δ^18^O record (RTP_O_s). There is clear evidence of the Mid-Pleistocene (MPT) critical transition, between 1.25 Ma and 0.8 Ma, during which a shift occurred from climate cycles dominated by a 40-kyr periodicity (due to obliquity) to 100-Kyr periodicity (due to eccentricity) dominated ones^73–77^. The 1.25 Ma date is particularly significant, since it is followed by an Increase in the amplitude of glacial–interglacial fluctuations.

A complementary RQA of the δ^18^O bulk carbonate record from U1308, which characterizes episodes of iceberg calving into the North Atlantic Ocean IRD released into the North Atlantic Ocean^25^, and therefore illustrates the dynamics of the Northern Hemisphere ice sheets (NHIS), yields similar dates to those obtained for the benthic δ^18^O record (see Suppl. Figure 3, Suppl. Tab. 1) ^72^. Indeed, one finds abrupt transitions at 2.75 Ma, at 1.5 Ma, at 1.25 Ma, at 0.9 Ma and 0.65 Ma. Finally, as opposed to the CENOGRID δ^13^C RP, U1308 δ^13^C RP shows a drifting pattern similar to that of benthic δ^18^O, with only 2 key transitions at 2.52 Ma and 0.48 Ma (see Suppl. Figure 3). Note that the 0.48 Ma transition does not have any equivalent in the benthic δ^18^O records.

## Discussion

Studying the same CENOGRID dataset, Boettner et al.^78^ identified 9 geological transitions at respectively 62.1 Ma, 55.9 Ma, 33.9 Ma, 23.2 Ma, 13.8 Ma, 10.8 Ma and 7.6 Ma. Four of them are indeed identical to those determined in the present study: 62.1 Ma, 55.9 Ma, 33.9 Ma and 13.8 Ma, the first two being preceded by a significant early warning signal^78^, which is instead absent in the case for the EOT key transition.

**Figure 5:**
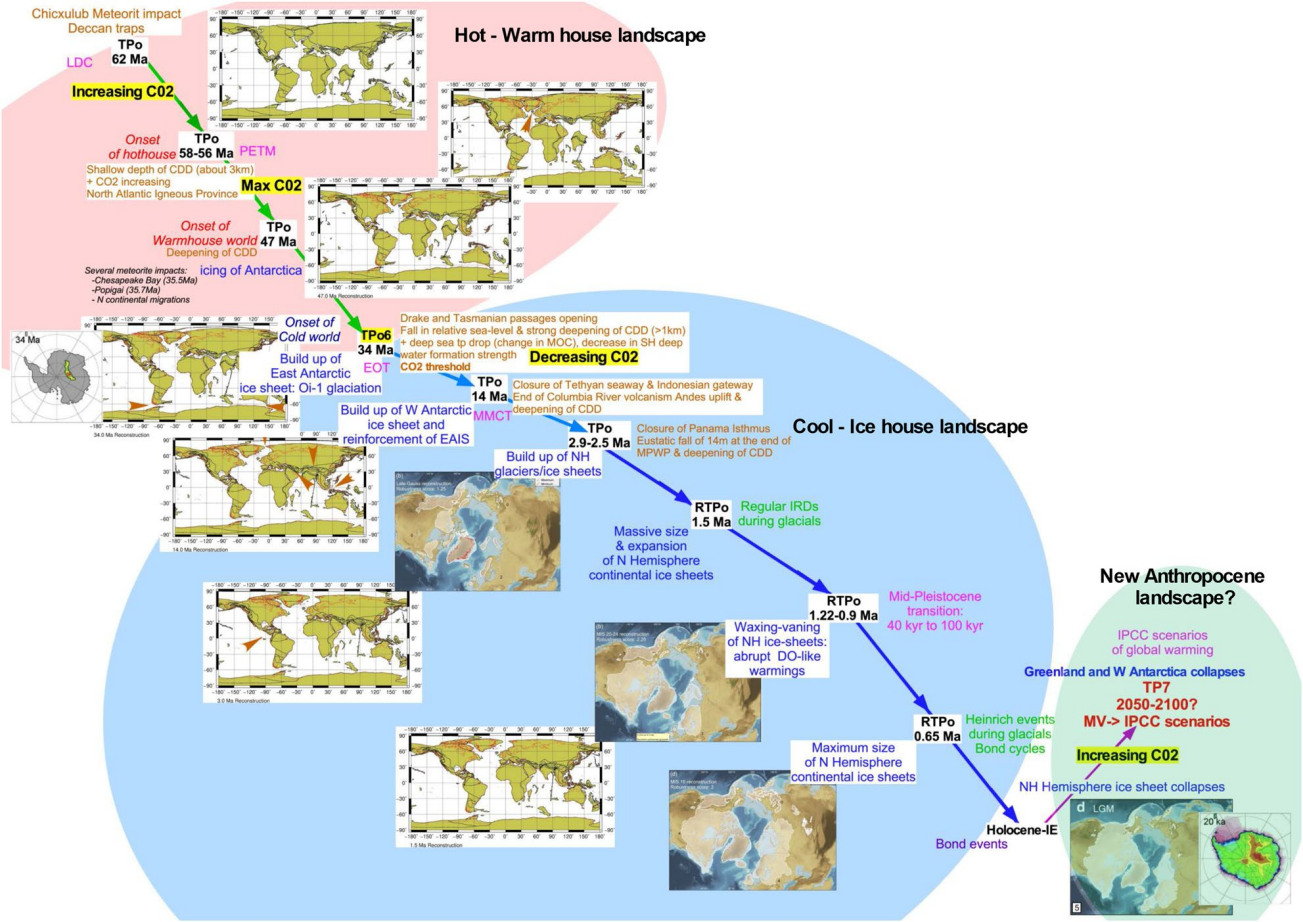
Evolution of the Earth Climate history among 2 different tipping dynamical landscapes and proposal for a potential third one. The first dynamical landscape, in light red, corresponds to the Hot-Warm House time interval. The second one, in light blue, represents the Cold-Ice house time interval. The third one, in light green, highlights the potential new dynamical landscape represented by the Anthropocene time interval. The different abrupt transitions identified in the present study are reported as TPos or RTPos to differentiate the major tipping points from the critical transitions characterizing transitions of lighter significance in the climate history. Various plate tectonic and ice sheet events are indicated and supported by maps of plate movements and North and South Hemisphere ice sheets. The Antarctica maps are from Pollard & DeConto^79^, Northern Hemisphere ice sheet maps are from Batchelor et al.^80^. The paleogeographic maps have been generated using the Ocean Drilling Stratigraphic Network (ODSN) plate tectonic reconstruction service: < https://www.odsn.de/odsn/services/paleomap/paleomap.html > . The red arrows on the tectonic maps indicate the key events that correspond to abrupt transition.

Based on the results of both the RQA and the KS test of the δ^18^O and δ^13^C time series considered in this study, and the bivariate analysis performed using the framework of the quasi-potential theory, we propose a succession of critical transitions as described in Fig. 5. The critical transitions TP_O_1 to TP_O_9 shaped the Earth climate towards the onset and development of the Southern ice sheets and the later build-up of the NHIS. TP_O_9 is followed by four more recent RTPs during the Quaternary, which steered the evolution of the ice sheets and of the climate as a whole until the present day. The climatic evolution during the Cenozoic until about 3 Ma seems to conform to a punctuated equilibrium framework, where the TP_O_s are associated with rapid transitions between rather different metastable modes of operation of the climate system. In particular, a key step that separates two rather diverse sets of climatic states occurred around 34 Ma at the EOT (TP_O_6). Without the major drop in GMSL, in CO_2_ concentrations, and in CCD, the Earth climate could have been different. However, after TP_O_6, the Earth climate entered new dynamical regimes marked by much lower CO_2_ concentrations, a lower GMSL, and a lower CCD. The remodeling of oceanic basins and mountain uplifts changed the marine and atmospheric circulations patterns, which played a crucial role in initiating and shaping the development of the NHIS.

Interestingly, the analysis of the δ^13^C time series identifies a different set of critical transitions, with the exception of the PETM (TP_O_2 and TP_C_3) and the EOT (TP_O_6 and TP_C_8), which are identified for both analyzed proxies. Looking into the bivariate pdf in the projected (δ^13^C, δ^18^O) space allows a better understanding of the nature and the origin of the TPs separately detected by studying the recurrence properties of the univariate time series. Indeed, we are in most cases able to associate both the TP_O_s and the TP_C_s to transitions across saddles of the effective quasi-potential. Clearly, some critical transitions might be more easily detectable when examining one time series rather than the other, because different proxies might be more sensitive to the active climatic processes, yet the approach taken here allows placing all TPs within a common ground. Additionally, TPs associated with faster processes and occurring between slower TPs correspond to transitions across smaller-scale saddles of the quasi-potential, hence revealing an analogy between the hierarchy of TPs and the multiscale nature of the quasi-potential.

More recently, variations in the extent and volume of the NHIS have contributed to the occurrence of the millennial variability marked by the Bond cycles, which have been best described during the last climate cycle. However, the onset of these cycles has been proposed to date back to 0.9 Ma^72^. Human activity is now rapidly pushing the Earth system towards the limits of its safe operating space associated with the occurrence of TPs; see also Rothman^9^ for a geochemical perspective focusing on the ongoing perturbation imposed on the marine carbon cycle. This concern is supported by actual observations impacting numerous tipping elements (see Lenton^14^) through very drastic tipping cascades^16^. This paves the way for a possible upcoming major transition, which might lead us to climate conditions that are fundamentally different from what has been observed in the recent or more distant past, and, at the very least, could bring us into a climate state with a much reduced or absent NHIS^38^. This potential major transition, leading to de facto irreversible changes for the climate and the biosphere, could become a boundary between the Cenozoic icehouse and a new, warmer and radically different climate state compared to Pleistocene conditions.

## Methods

### Recurrence plots and Kolmogorov-Smirnov test

The Kolmogorov–Smirnov (KS) test is a robust method for accurately detecting discontinuities in a particular time series and is therefore a very precise way for determining the timing of abrupt transitions. Our method, described by Bagniewski et al.^26^ is modified from the two-sample KS test and has been successfully applied to various geological time series^24,25,69^. The method uses the KS statistic, *D*_KS_, to compare two sample distributions taken before and after a potential transition point within a sliding window. If the samples do not belong to the same continuous distribution, i.e., *D*_KS_ is greater than a predefined threshold, a transition point is identified.

This classic two-sample KS test is augmented by additional criteria to refine the results and pinpoint significant abrupt transitions indicative of a true climatic shift. This involves discarding transitions below a rate-of-change threshold since smaller changes in the time series might be due to data errors or short-term variability within intervals shorter than the proxy record's sampling resolution. Furthermore, as the frequency with which the KS test detects transitions largely depends on the chosen window length, *D*_KS_ is calculated for different window length, within a range that corresponds to the desired time scale at which a given paleorecord is to be investigated. The analysis starts by identifying transitions using the longest window, which has the largest sample size and thus carries greater statistical significance. Subsequently, the method incorporates transitions detected using shorter windows to capture transitions occurring on shorter time scales. For more details see Bagniewski et al.^26^, Rousseau et al.^72^.

To gain further insight into the climate story revealed by proxy records, we performed an analysis based on recurrence plots (RPs), first introduced by Eckmann^81^ in the study of dynamical systems and later popularized in the climate sciences by Marwan et al. ^53,82^. The RP for a time series $\left\{ {{\text{x}}_{{\text{i}}} :{\text{ i }} = { }1, \ldots ,{\text{N}}} \right\}$ is constructed as a square matrix in a Cartesian plane with one copy $\left\{ {{\text{x}}_{{\text{i}}} } \right\}$ of the series on the abscissa and another copy $\left\{ {{\text{x}}_{{\text{j}}} } \right\}$ on the ordinate, with both axes representing time. A dot is entered into a position (i, j) of the matrix when ${\text{x}}_{{\text{j}}}$ is sufficiently close to ${\text{x}}_{{\text{i}}}$. For the details—such as how “sufficiently close” is determined—we refer to Marwan et al. ^50^.

The square matrix of dots that is the visual result of RP exhibits a characteristic pattern of vertical and horizontal lines, indicating recurrences. These lines sometimes form clusters that represent specific periodic patterns. Eckmann^81^ distinguished between large-scale *typology* and small-scale *texture* in the interpretation of RPs. The most interesting typologies in RP applications are associated with recurrent patterns that are not strictly periodic and are thus challenging to detect by purely spectral approaches to time series analysis. RPs offer an important advantage by enabling a visual identification of nonlinear relationships and dynamic patterns that characterize high-dimensional systems subject to time-dependent forcing, such as the climate system. However, in periodically forced systems, the recurrence structure is dominated by repetitive, periodic patterns rather. This may pose challenges when attempting to identify meaningful transitions or regime shifts in climate at time scales strongly affected by orbital forcing.

Marwan et al.^82^ extensively discussed the various measures used to objectively quantify the typologies observed in RPs. Collectively known as recurrence quantification analysis (RQA), these measures include the Recurrence Rate (RR), which represents the probability of a specific state recurring within a given time interval. The RR is calculated by determining the density of recurrence points along the diagonal of the RP within a sliding window. Since low RR values correspond to an unstable behavior of the system, the minima of the RR may be used to identify abrupt transitions. Here we follow the approach by Bagniewski et al.^26^ and select local minima based on their prominence.

### Quasi-potential, Melancholia states, and critical transitions

Traditionally, tipping points are schematically represented as being associated with the bifurcation occurring for a system described by a one-dimensional effective potential when a change in the value of a certain parameter leads to a change in the number of stable equilibriums. Hence, conditions describing the nearing of a tipping point can be related to the presence of slower decay of correlations (critical slowing down). This viewpoint, while attractive, suffers from many mathematical issues due to the fact that the true dynamics of the system occurs in a possibly very high dimensional space. Tantet et al.^83,84^ and Santos Gutierrez & Lucarini^85^ have introduced a mathematically rigorous framework for the occurrence of tipping points that clarifies the link between rate of decay of correlations, sensitivity of the system to perturbations, and robustness of the unperturbed dynamics.

Here, we wish to take a different angle on the problem. Instead of focusing on the individual tipping points, we attempt to capture the global stability properties of the system. We take inspiration from the application of the Waddington epigenetic landscape to describe morphological evolution^42–46^ and from the theory of punctuated equilibrium^49,50^, which associates periods of stasis (characterized by relatively stable morphology) with the emergence of new species through abrupt changes (named cladogenesis) from a previous species. Lucarini & Bodai^39,40^ proposed describing the global properties of the climate system using the formalism of quasi-potential^41^. Roughly speaking, assuming that the system lives in ${\mathbb{R}}^{N}$, and its dynamics is described by a stochastic differential equation the probability that its state is within the volume $d\vec{x}$ around the poin $\vec{x} \in {\mathbb{R}}^{N}$ is given by $P\left( {\vec{x},d\vec{x}} \right) = \rho \left( {\vec{x}} \right)d\vec{x}$ where $\rho \left( {\vec{x}} \right) \approx e^{{ - \frac{{2{\Phi }\left( {\vec{x}} \right)}}{{\varepsilon^{2} }}}}$ is the probability distribution function (pdf) and ${\Phi }\left( {\vec{x}} \right)$ is the quasipotential.

The function ${\Phi }\left( {\vec{x}} \right)$ depends in a nontrivial way on the drift term and noise law defining the stochastic differential equation. This setting generalizes the classical energy landscape and applies to a fairly large class of stochastic dynamical systems. One can see the dynamics of the system as being driven towards lower values ${\Phi }\left( {\vec{x}} \right)$ (plus an extra rotational effect that is typical of non-equilibrium systems), while the stochastic forcing noise makes sure that the system is erratically pushed around. Hence, the minima of ${\Phi }\left( {\vec{x}} \right)$ correspond to local maxima of the pdf, and the saddles (which coincide for both ${\Phi }\left( {\vec{x}} \right)$ and $\rho \left( {\vec{x}} \right)$) coincide with the Melancholia (M) states^34^. Such M states are unstable states of the system that live at the boundary between basins of attraction and are the gateways for the noise-induced transitions between competing stable states. Margazoglou et al.^37^ applied this method to the investigation of the metastability properties of an intermediate complexity climate model and suggested that the presence of decorations of the quasi-potential at different scales could be interpreted as being associated with a hierarchy of tipping points. Indeed, passing near M states is intimately associated with the occurrence of critical transitions. Hence, the construction of the quasi-potential ${\Phi }\left( {\vec{x}} \right)$ can be seen as the structural counterpart of the investigation of the time-evolution of the system and of its critical transitions. Zhou et al.^86^ give a complete overview of different methods applied to perform such an analysis.

## Data and materials availability

All data generated by the present study from the main text or the supplementary materials will be submitted to PANGAEA data repository. U1308 marine data are available at https://doi.org/10.1594/PANGAEA.871937 (Hodell and Channell, 2016b). CENOGRID data are available at https://doi.org/10.1594/PANGAEA.917503 (Westerhold, 2020).

## Supplementary Information

Supplementary Information 1.

Supplementary Information 2.
