# Supplementary material for: A punctuated equilibrium analysis of the climate evolution of cenozoic exhibits a hierarchy of abrupt transitions
Source: Sci Rep. 2023 Jul 12;13:11290. doi: 10.1038/s41598-023-38454-6 (PMC10338496; doi:10.1038/s41598-023-38454-6)
Supplement: Supplementary file 1 — Supplementary Information 1. [file 41598_2023_38454_MOESM1_ESM.pdf]

A Punctuated Equilibrium Analysis of the Climate Evolution of Cenozoic exhibits a Hierarchy of Abrupt Transitions

Denis-Didier Rousseau, Witold Bagniewski, Valerio Lucarini

Appendices

A Historical Account of the Critical Transitions

In addition to the Chicxulub meteor impact, which injected a considerable amount of CO_2 into the atmosphere[1,2], Deccan traps were already spreading at the beginning of the Cenozoic, contributing to the release of massive amount of CO_2[3]. CO_2 concentrations continued to rise until reaching about 500 ppmv, coinciding with a very active period of the North Atlantic Igneous Province around 58 Ma – 56 Ma (TP_O2 and TP_O3), which was associated with the opening of the North Atlantic Ocean[4]. During this time, the Northern Hemisphere plates were connected and did not experience the present Arctic conditions, allowing faunal and vegetal dispersion. In contrast, other plates were undergoing reorganization, such as India moving northeastward towards the Asian continent. Equatorial Pacific carbonate compensation depth (CCD) reached its minimum value a bit later at about 54 Ma when CO_2 concentrations were at a maximum for the whole Cenozoic, above 1,100 ppmv[5]. The decrease in GMSL and the CCD at TP_O4 (about 40 Ma) has been interpreted as the start of the icing of Antarctica through dating mountain glacier deposits using K-Ar dating of lava flows[6], as well as other glacial evidence, i.e. from the Gamburtsev subglacial mountains[7]. During this period, the Northern Hemisphere plates remained connected. By TP_O4 India was approaching the Asian plate while Northern Hemisphere plates remained connected, allowing continental migrations of mammals at high latitudes[8,9]. Around 34 Ma, the EOT (TP_O6) is associated with the opening (in several steps) of the Drake and Tasmanian passages[10], which led to a drastic change in the global ocean circulation. This resulted in a reduced strength of deep water formation in the Southern Hemisphere, and a decline in deep-sea temperatures, accompanied by a significant drop in relative sea level and a decrease in CCD (see Fig. 2). Based on paleoaltimetry estimates using oxygen isotopes, Rowley & Currie[11] indicate that the Tibetan Plateau had an elevation of about 4000m, favoring therefore the physical weathering of rocks, resulting in the consumption of CO_2, and the enhanced burial of carbon through high sedimentation rates in the nearby seas. This may have contributed to the major threshold in the variation of the CO_2 concentration[12], which also drops very strongly[5] (Fig. 2).

Recent analysis of the evolution of sea surface temperatures in the North Atlantic Ocean during the Eocene-Oligocene Transition (EOT) suggests that the cooling during this period was primarily triggered by a decrease in CO2 concentration, with paleogeographic changes playing a secondary role[13]. Comparisons between marine and terrestrial data and models reveal a more abrupt decrease in pCO2 in marine proxies, as evidenced by the decrease in the CCD, contrasting with a more gradual decrease in terrestrial indicators. The gradual decrease observed in terrestrial proxies remains a topic of debate. Additionally, these studies suggest that paleogeographic changes played a significant role in initiating the Atlantic Meridional Overturning Circulation (AMOC), which became an important ocean circulation pattern in the growth of polar ice sheets (Hutchinson et al. [14], and references herein).

As mentioned, The EOT transition is the major boundary between two different climate landscapes dominated by intensive plate tectonics and strong volcanism for the older one, and by major ice sheet coverage in both Hemispheres, with still very active plate tectonics (closure of seaways, orogenies), for the younger one[9]. Following the EOT, the climate experienced the

build-up of the East Antarctic ice sheet (Oi-1 glaciation), which can be regarded as the onset of the cold world in which we are presently living. During this time, India has almost ended its transfer to the Asian plate. Between TP_O 6 and TP_O 7, i.e., 34 Ma and 14 Ma respectively, the East Antarctic ice sheet underwent waxing and waning with several major glaciations occurring before the 17 Ma to 14.5 Ma interval, characterized by rising sea levels, a severe shoaling episode of CCD, and higher CO_2 concentrations (Fig. 2). Such high CO_2 concentrations may have been fueled by the Columbia River major volcanism, which ended by TP_O 7 at about 14 Ma[3]. Plate tectonics still played a very active role, with the closure of both the Indonesian gateway and the Tethyan seaway, and contributing to the start of the development of the Mediterranean [75]. Eurasia is now separated from North America and Greenland, India colliding with the Asian continent, and the Andes are uplifting, thus modifying the geometry of the marine basins and the global oceanic circulation. West Antarctica is beginning to form while the East Antarctic ice sheet continues to strengthen and expand. TP_O8 at about 9 Ma sees a strong lowering of the GMSL and of the CO_2 concentrations (Fig. 2). TP_O9 is associated with a final major tectonic event corresponding to the closure of the Panama Isthmus, which, as a result of shutting down the exchange of water between the two oceans, led to re-routing large-scale flows in both the Pacific and the Atlantic Ocean, and configured a oceanic circulation that is very similar to present day[15]. This is associated with a significant decrease in the global sea level, a deepening of the CCD, and lower CO_2 concentrations (Fig. 2). These conditions will shape the Earth climate history during the Quaternary, leading to the build-up of the Northern Hemisphere glaciers and ice sheets. It is worth noting that all the major transitions that have been identified during the interval between 66 Ma and 2.9 Ma - 2.5 Ma are associated with the build-up, waxing, and waning of the Northern and Southern Hemisphere ice sheets.

References

1. O'Keefe, J. D. & Ahrens, T. J. Impact production of C02 by the Cretaceous/Tertiary extinction bolide and the resultant heating of the Earth. *Nature* 338, 247–249 (1989).
2. Lomax, B., Beerling, D., Upchurch, G. & Otto-Bliesner, B. Rapid (10-yr) recovery of terrestrial productivity in a simulation study of the terminal Cretaceous impact event. *Earth Plane. Sci Lett.* 192, 137–144 (2001).
3. Scotese, C. R., Song, H., Mills, B. J. W. & van der Meer, D. G. Phanerozoic paleotemperatures: The earth's changing climate during the last 540 million years. *Earth-Science Reviews* 215, (2021).
4. Jolley, D. W. & Bell, B. R. The North Atlantic Igneous Province: Stratigraphy, Tectonic, Volcanic, and Magmatic Processes. in (Geological Society of London, 2002).
5. Beerling, D. & Royer, D. Convergent Cenozoic CO2 history. *Nature Geoscience* 4, 418–420 (2011).
6. Birkenmajer, K. *et al.* First Cenozoic glaciers in west Antarctica. *Polish Polar Research* 3-12-3–12 (2005).
7. Rose, K. *et al.* Early East Antarctic Ice Sheet growth recorded in the landscape of the Gamburtsev Subglacial Mountains. *Earth and Planetary Science Letters* 375, 1–12 (2013).
8. Janis, C. Tertiary mammal evolution in the context of changing climates, vegetation, and tectonic events. *Annual Review of Ecology and Systematics* 24, 467–500 (1993).
9. Torsvik, T. H. & Cocks, L. R. M. *Earth history and palaeogeography*. (Cambridge University Press, 2016).
10. Lagabrielle, Y., Godderis, Y., Donnadieu, Y., Malavieille, J. & Suarez, M. The tectonic history of Drake Passage and its possible impacts on global climate. *Earth and Planetary Science Letters* 279, 197–211 (2009).

11. Rowley, D. & Currie, B. Palaeo-altimetry of the late Eocene to Miocene Lunpola basin, central Tibet. *Nature* 439, 677–681 (2006).
12. Rae, J. W. *et al.* Atmospheric CO2 over the past 66 million years from marine archives. *Annual Review of Earth and Planetary Sciences* 49, 609–641 (2021).
13. Sliwinska, K. *et al.* Sea surface temperature evolution of the North Atlantic Ocean across the Eocene-Oligocene transition. *CLIMATE OF THE PAST* 19, 123–140 (2023).
14. Hutchinson, D. *et al.* The Eocene-Oligocene transition: a review of marine and terrestrial proxy data, models and model data comparisons. *CLIMATE OF THE PAST* 17, 269–315 (2021).
15. Lunt, D., Valdes, P., Haywood, A. & Rutt, I. Closure of the Panama Seaway during the Pliocene: implications for climate and Northern Hemisphere glaciation. *Climate Dynamics* 30, 1–18 (2008).
