# Supplementary material for: A punctuated equilibrium analysis of the climate evolution of cenozoic exhibits a hierarchy of abrupt transitions
Source: Sci Rep. 2023 Jul 12;13:11290. doi: 10.1038/s41598-023-38454-6 (PMC10338496; doi:10.1038/s41598-023-38454-6)
Supplement: Supplementary file 2 — Supplementary Information 2. [file 41598_2023_38454_MOESM2_ESM.pdf]

A Punctuated Equilibrium Analysis of the Climate Evolution of Cenozoic exhibits a Hierarchy of Abrupt Transitions

Denis-Didier Rousseau, Witold Bagniewski, Valerio Lucarini

Supplementary Information

Comparison between GMSL, CCD and CO_2 concentration from CENOGRID (past 66 Myr)

We provide here a more detailed discussion of TP_O 1-9 in relation to reconstructed global mean sea level (GMSL), Pacific carbonate compensation depth (CCD), and CO_2 concentration. The interval between 66 Ma and 63 Ma (TP_o1) shows a relative stable GMSL above +60m. It is followed by a lowering by about 70m over several steps between TP_o1 at 63 Ma and TP_o3 at 56 Ma, punctuated by an abrupt increase of 52m at TP_o2 around 58 Ma, and another increase of 28m corresponding to the short but intense Paleocene-Eocene Thermal Maximum (PETM – Fig. 2a) warming. Subsequently, GMSL raises by about +40m between TP_o2 at 58 Ma and 55 Ma. The interval between 55 Ma and 48 Ma, the Early Eocene climatic optimum (EEOC – Fig. 2A), is characterized by a relatively high GMSL of about 66 m above the present mean sea level, which is associated with the occurrence of hyperthermal conditions at 58 Ma, 57 Ma, and 53 Ma. Between 52 Ma and TP4 at about 47 Ma, CCD reaches the shallowest depth of the record, about 3000m [1], associated with the highest CO2 concentrations estimated above 1100 ppmv by Beerling and Royer [2] – Fig. 2B,C). A strong decrease in GMSL of about 30m occurs between 48 Ma and 46 Ma while it remains relatively stable at about +42m between 46 Ma and TP5, at 40 Ma. It is however punctuated by two short events, first a lowering of about 25m between 42 Ma and 40.5 Ma, and next by a strong increase of about 40m between 40.5 Ma and 40 Ma (TP_o5) corresponding to the Middle Eocene Climatic optimum (MECO – Fig. 3A). A two-step lowering of about 35m of the GMSL occurs between 40 Ma (TP_o5) and 36 Ma, followed by a gradual increase of about 25m between 36 Ma and 34.5 Ma just before the Eocene-Oligocene Transition (EOT – Fig. 3A). Between 46 Ma and 34 Ma (TP_o6), the CCD is strongly oscillating with numerous deepening and shoaling events of 500 to 1000 m in magnitude and shoaling ones, corresponding to carbonate accumulation episodes. These oscillations in the CCD occurred during an interval indicating still high CO2 concentrations, roughly

above the 750 ppmv considered as the Antarctic glaciation threshold [3], and marked by a relative minimum at about TP_o5 at 40 Ma (Fig. 3B,C). This first interval of the GMSL, which ends with a strong deepening of about 1000m, is associated with a strong decrease in CO2 concentration and an almost completely ice free Earth, with no major ice sheet in either the southern or northern hemisphere. This is deduced primarily from the high GMSL, mostly remaining above 12m (Fig. 2A), which would correspond to the mutual contribution of the Greenland and the West Antarctic ice sheets. Additionally, high CO2 concentrations estimates are in agreement with a CCD generally lower than 4000 m (Fig 2B,C).

The second main interval shows a completely different scenario with the GMSL varying between +30m and -80m without considering the late Quaternary interval, and much lower CCD and CO2 concentration (Fig. 2B,C). First the strong decrease in the GMSL at EOT reaches negative values of about -25m at about 33.5Ma. It is interpreted as evidence of the first continental scale Antarctic ice sheet (first Oligocene isotope maximum - Oi1 – Fig. 2A) [4]. After a return to values similar to present mean sea level, about +2m, between 32 Ma and 30 Ma, a new decrease of about 25m in GMSL occurs between 29.5 Ma and 27 Ma corresponding to another continental scale Antarctic ice sheet extent labeled Oi2 (Fig. 2A). After a two-step increase in GMSL of about 40m between 27 Ma and 24 Ma, a new sharp decrease of about 40m is noticed at about 23 Ma. It corresponds to the Middle Oligocene Maximum (Mi1 – Fig. 2A), another Antarctic ice sheet wide expansion [4,5]. From TP_o6, at 34 Ma, and 23 Ma, the CO2 concentration decreases associated with a deepening trend in the CCD down to about -4600m. From 23 Ma until about 19 Ma, GMSL shows oscillations but with lower values than present day at about -20m, whereas from 19 Ma until 17 Ma, GMSL increases by about 50m to indicate high values around +30 m above present day value (Fig. 2A). The CCD indicates about 600 m shoaling which lasted around 2.5 Myr linked to high estimates of CO2 concentration from paleosols and stomata [2] (Fig. 2B,C). This strong increase corresponds to the Miocene Climatic optimum (MCO – Fig. 2A) between 17 Ma and TP_o7 at 13.9 Ma, which is the last interval during which GMSL reaches such high values higher than +20m above the present day ones (Fig. 2). The interval between TP_o7 at 13.9 Ma and 13 Ma corresponds to the Middle Miocene

transition during which GMSL once more decreases significantly by about 35m. Such lowering is associated with the growth of the East Antarctic ice sheet to near its present state, remaining a perennial ice body that is thereafter impacting the Earth climate [6,7]. Although GMSL remains relatively stable between 13 Ma and 12 Ma, another strong decrease, again of about 30m, occurs between TP_o8 at about 9 Ma and 8.5 Ma, associated with the strongest deepening recorded by the CCD, around 4800m. GMSL increases again by about 20m until 7.5 Ma to remain relatively stable until 5.5 Ma when GMSL increases by about 20m between 5.5 Ma and 3.5 Ma, corresponding to the Pliocene Climatic Optimum (PCO – Fig. 2A). Between 3.5 Ma until TP_o9 at about 2.7 Ma GMSL shows a sharp decreasing trend of about 35m (Mi2a, 3, 4 – Fig. 2A). This is associated with the development of the large northern ice sheets between 2.9 Ma (TP_o9a) and 2.5 Ma (TP_o9b – Fig. 2), especially the Laurentide ice sheet corresponding to about 50m decrease of GMSL with regards to the present day value (Fig. 2A). From TP_o8 at about 9 onward, CCD shows significant fluctuations, although it remains at around 4500m with two strong deepening events at about TP_o9a 2.9 Ma and TP_o9b (Fig. 2) at 2.5 Ma.

In another global sea level reconstruction[8], several major thresholds were identified agreeing with the Miller et al [10] reconstruction and our present analysis. This reconstruction estimates the EOT global sea level drop at 34 Ma (TP_o6) as about 30m, while previous reconstructions by Houben et al. [9], and by Miller et al. [10] found a decrease of 70m-80m. Miller et al [10] also estimate this drop in global sea level being associated with a 2.5°C cooling interpreted previously as above the onset of the Antarctic ice sheet glaciation. Rohling et al. [8] also identify 14 Ma (TP_o7) threshold as the end of the last intermittently ice free period in the Earth history of the last 40 Ma with only the southern hemisphere ice sheets impacting the Earth climate. Miller et al. [10] indicates a 35m lowering at that particular transition. Indeed Rohling et al. [8] indicate a slight negative sea level shift at around 10 Ma (TP_o8), of about 10m,, which they interpret as the onset of partial or ephemeral northern Hemisphere ice bodies with two other sea level thresholds at about 3 Ma (close to TP_o9a) and 2.75 Ma (TP_o9b), also observed in Miller et al. [10]. These two key dates correspond to respectively to the first major iceberg calving in the Nordic Seas [11] and from the Laurentide ice sheet [12] although this interpretation was rejected by Naafs et al. [13] who instead attributed the IRD

signature to Greenland and Fennoscandian glaciers. Hodell and Channell [14] identified the first occurrence of iceberg calving in North Atlantic at about 2.75 Ma from the analysis of the $\delta^{18}O$ of benthic bulk carbonate in core U1308 (Supp. Fig. 3), a date identified as an abrupt transition in the RR analysis of both $\delta^{18}O$ of benthic foram and bulk carbonate [15]. All along the past 66 Ma, GMSL has been varying between average values of +38. m ± 15 m above the present day value during the hot world interval prior to TP_o6 at 34 Ma, and of -3.5m ± 13 m from 34 Ma until the present day during the cold world interval (Tab. 2).

The past 3.3 Myr.

We provide here a more detailed discussion of the past 3.3 Myr. The first date, TP_o9b detected by RQA, is interpreted as corresponding to the earliest occurrence of IRD in the North Atlantic. This occurrence characterizes the presence of Northern Hemisphere coastal glaciers large enough to calve icebergs in the ocean, and the melting of these icebergs is likely to have impacted the oceanic circulation. Naafs et al.[13], however, reported the occurrence of weak IRD events in the late Pliocene that they attributed mainly to Greenland and Fennoscandian glaciers. Nevertheless, such interpretation points to nevertheless smaller ice sheets over these regions than during the later Quaternary, when North American ice sheets were considerably larger. The interval TP_o9a, at 2.8 Ma, to RTP_o2, at 1.2 Ma, shows glacial–interglacial sea level variations of about 25–50 m below the present day. The CO2 concentrations varied between 270 ppmv and 280 ppmv during interglacials and between 210 ppmv and 240 ppmv during glacials, with a decreasing trend of about 23 ppmv over this 1.4-Myr–long interval [16].

The second date, RTP_o1, at 1.55 Ma, corresponds to an increased amplitude in ice volume variations between glacial minima and interglacial optima. This second step shows the permanent occurrence of ice-rafted events during glacial intervals in the record (Suppl. Fig. 3), therefore indicating an amplified relationship of climate variations with Northern Hemisphere ice sheets. The increase in IRD variability and magnitude since RTP_o1, however, shows that distinct, faster processes have to be considered than those due to slow changes in Earth's orbital parameters; see again Fig. 4.

The third date, RTP_o2, at 1.25 Ma, close to the MIS22–24 $\delta^{18}O$ optima, shows increased continental ice volume in the Northern Hemisphere [17], but also more stability

in the East Antarctic ice sheet in the Southern Hemisphere [18]. In parallel, evidence of a major glacial pulse recorded in Italy's Po Plain, as well as in ^{10}Be-dated boulders in Switzerland, is interpreted as marking the onset of the first major glaciation in the Alps [19,20].

After RTP_o2, at 1.25 Ma, the sea level decreased to about 70–120 m below the present day, while the CO2 concentrations varied between 250 ppmv and 320 ppmv during interglacials and between 170 ppmv and 210 ppmv during glacials [21]. Similar variations were determined by Seki et al. [22], although pCO2 changes that occurred before the time reached by ice core records are associated with high uncertainties in both dating and values. The sawtooth pattern of the interglacial– glacial cycles [23] becomes noticeable at 0.9 Ma. At about the same time, the synthetic Greenland $\delta^{18}O$ reconstruction indicates the occurrence of millennial variability expressed by DO-like events [24].

Finally, RTP_o3, at 0.65 Ma, marks the end of the transition from the Lower and Mid-Pleistocene interval — characterized by 41-Kyr–dominated cycles and smaller 23-Kyr ones — to the Upper Pleistocene, with its 100-Kyr–dominated cycles; see Fig. 4. The sawtooth pattern of the interglacial–glacial cycles is well established during this final interval, in contradistinction with the previous, more smoothly shaped pattern that appears to follow the obliquity variations. The global ice volume is maximal, exceeding the values observed earlier in the record, especially due to the larger contribution of the Northern American ice sheets. The latter now have a bigger impact on Northern Hemisphere climate than the Eurasian ice sheets [17]. The IRD event intensity and the frequency of occurrence increase [25] as well (Suppl. Fig. 3), leading to the major iceberg discharges into the North Atlantic named Heinrich events (HEs); see [26–29]. The interval of 1 Ma – about 0.4 Ma (RTP_o4) is also the interval during which Northern Hemisphere ice sheets reached their southernmost extent [17]. Applying Mg/Ca transfer functions, Elderfield et al. [30] have estimated that the past 0.4 Myr water temperatures have been the highest the past 1.2 Myr, supporting the local temperature variations deduced from the Antarctic ice cores [31]

Figure S1: KS test and Recurrence Quantification Analysis (RQA) of CENOGRID benthic $\delta^{13}C$. A) KS test identifying abrupt transitions towards warmer conditions in red and cooler or colder conditions in blue; B) Recurrence plot (RP), and C) Recurrence rate (RR). The pink crosses and vertical green lines indicate the abrupt transitions (Table) detected by the RQA.

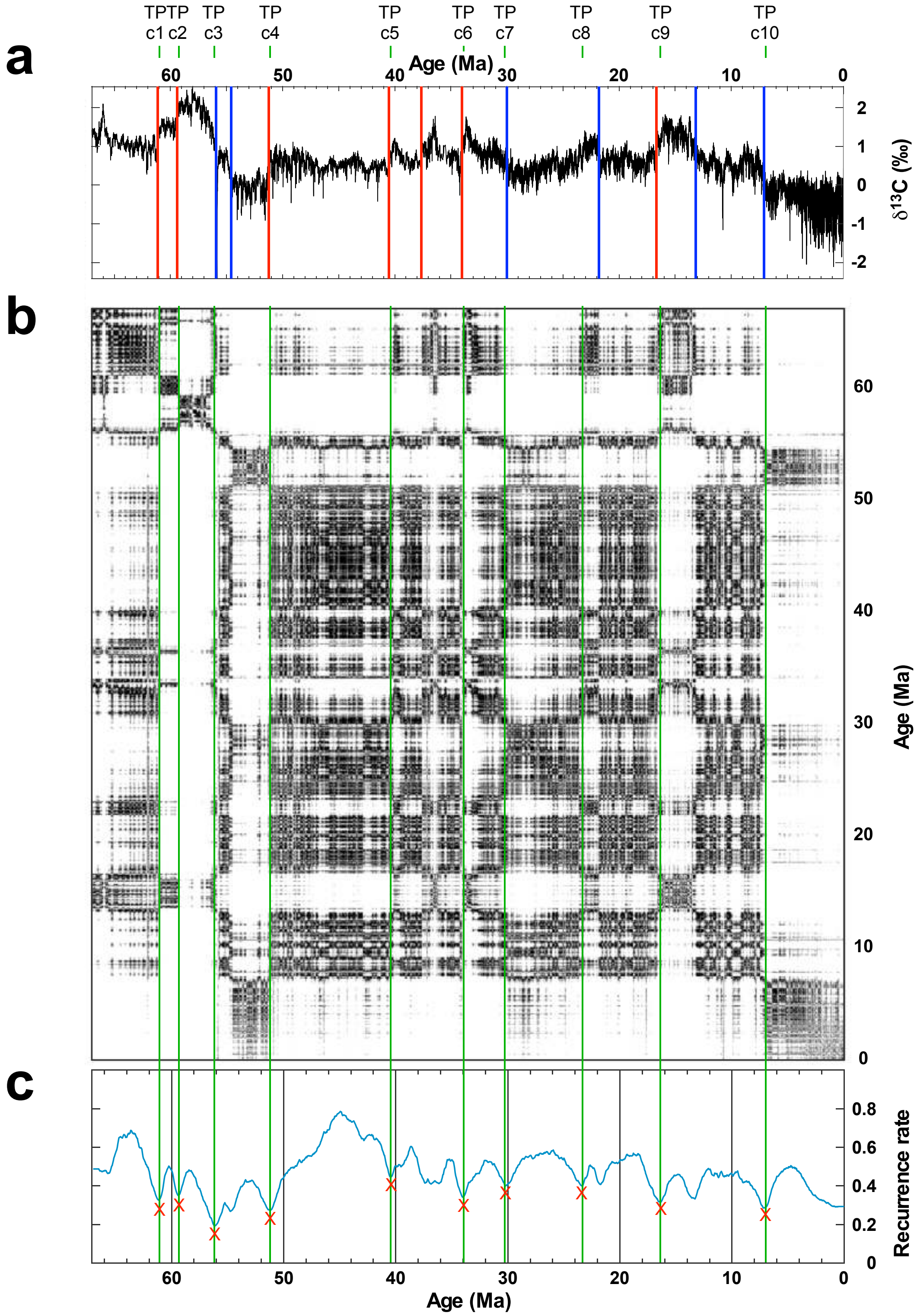

Figure S2: RQA of U1308 benthic $\delta^{13}C$. a) Time series in Ma; b) RP; and c) RR. Crosses as in Fig. S1.

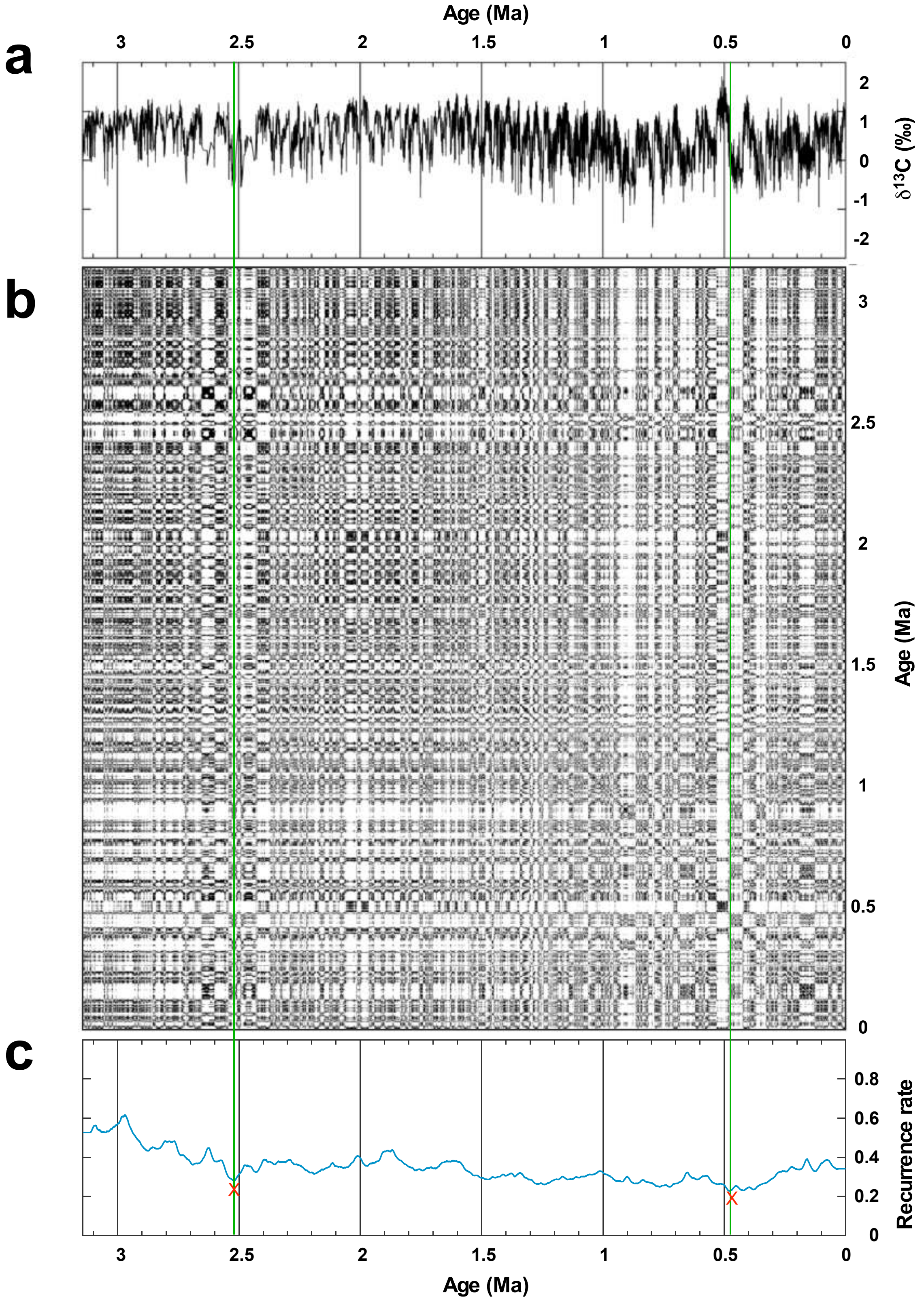

Figure. S3: RQA of U1308 bulk carbonate δ180. A) Time series in Ma of benthic $\delta^{18}O$ in blue and $\delta^{18}O$ bulk carbonate in green; b) RP; and c) RR). The pink crosses and vertical lines indicate the abrupt transitions (Table) detected by the RQA.

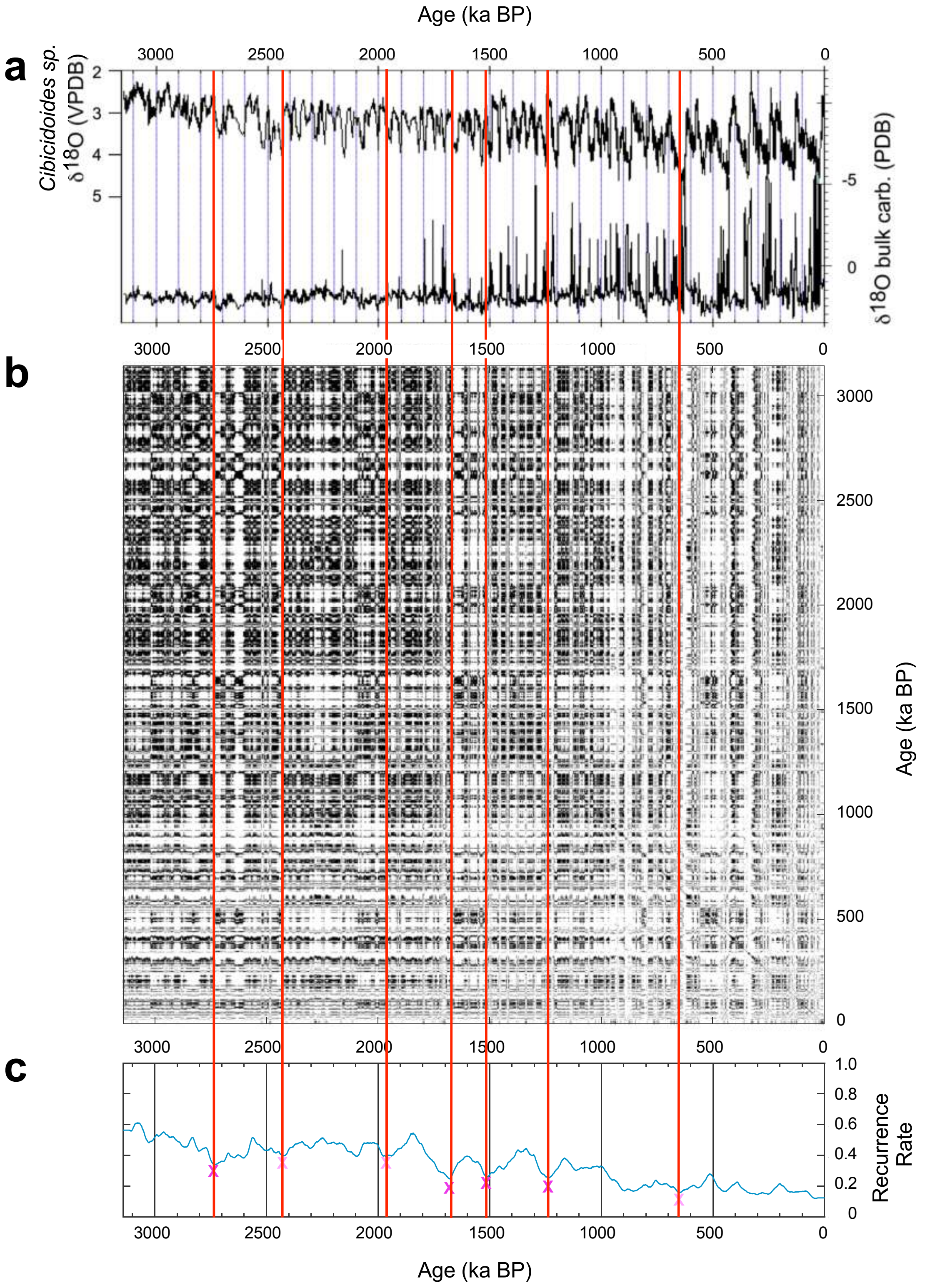

Table S1: Recurrence Quantification Analyses (RQA) of CENOGRID benthic $\delta^{18}O$ and $\delta^{13}C$, and U1308 benthic $\delta^{18}O$, $\delta^{13}C$, and bulk carbonate $\delta^{18}O$. For each, listed are the dates of the abrupt transitions, the corresponding RR prominence values used to select the abrupt transitions, and the significance of those transitions. X denotes prominence values above the RR standard deviation, while $ denotes values below the standard deviation. Ages are in Ma for the CENOGRID and in ka BP for the U1308 records respectively.

CENOGRID δ18O, window: 1-6 Ma			CENOGRID δ13C, window: 1-6 Ma			U1308 δ18O, window: 60-250 ka			U1308 δ13C, window: 60-250 ka			U1308 bulk carbonate δ18O, window: 60-250 ka		
Ma BP	RR prominen	Significance	Ma BP	RR prominen	Significance	ka BP	RR prominen	Significance	ka BP	RR prominen	Significance	ka BP	RR prominen	Significance
33,85	0,41570578	X	56,15	0,49582588	X	2524	0,23796191	X	477	0,16393608	X	2732	0,20363551	X
47	0,33114787	X	34	0,24468463	X	1510	0,14169216	X	2521	0,15914759	X	1681	0,19748231	X
14,05	0,31556165	X	7,15	0,21940684	X	354	0,1239728	X	-	-		1510	0,13491356	X
62,65	0,23176521	X	61,15	0,17631944	X	614	0,11154923	X	-	-		1234	0,12752732	X
39,85	0,18154617	X	16,4	0,17583585	X	1248	0,08692142	X	-	-		1966	0,12498718	$
56,05	0,17128033	X	23,4	0,16939465	X	2925	0,07068202	$	-	-		653	0,12224033	$
58,05	0,12919749	X	40,45	0,16112753	X	-	-		-	-		2421	0,11758806	$
9,7	0,0862254	$	51,2	0,15785367	X	-	-		-	-		-	-	
-	-		59,4	0,13334579	X	-	-		-	-		-	-	
-	-		30,2	0,13193157	X	-	-		-	-		-	-	

Table S2: Statistics of the 66-34 Ma and 34 Ma-present intervals, from top to bottom: the Global Mean sea level (GMSL) in meters from Miller et al. [10], the CO2 concentration in ppmv from Beerling and Royer [2], and for the CCD depth in meters from Palike et al. [1].

Miller et al. (2020)	Age_cal Ma BP	Sea level (m)		Age_cal Ma BP	Sea level (m)
Minimum	0,98	-33,00		33,68	-1,90
Maximum	33,66	33,20		64,82	77,30
Points	1635,00	1635,00		1558,00	1558,00
Mean	17,32	-3,49		49,25	38,47
Median	17,32	-3,80		49,25	36,00
Std Deviation	9,44	12,90		9,00	14,85

Beerling & Royer (2011)	Age (Ma)	CO2 (ppm)		Age (Ma)	CO2 (ppm)
Minimum	0,00	80,00		34,00	100,00
Maximum	33,60	1232,00		65,00	1868,00
Points	289,00	289,00		81,00	77,00
Mean	14,29	329,81		49,17	626,96
Median	14,10	271,00		54,00	574,00
Std Deviation	8,39	164,47		9,73	311,79

Pälike et al. (2012)	Age (Ma)	eq CCD (m)		Age (Ma)	eq CCD (m)
Minimum	0,00	4100,00		33,75	2800,00
Maximum	33,50	4900,00		52,25	4300,00
Points	135,00	135,00		75,00	75,00
Mean	16,75	4586,30		43,00	3518,67
Median	16,75	4600,00		43,00	3500,00
Std Deviation	9,78	153,01		5,45	408,92

Table S3: Summary of the GMSL, CCD and CO2 concentration trends at the identified abrupt transitions TP1 to TP10.

	TP1	TP2	TP3	TP4	TP5	TP6	TP7	TP8	TP9	TP10
**GMSL**	↘	↗	⇘	↘	↗	⬊	↘	↘	↘	↘
**CCD**	-	-	-	↘	⇗	⬊	↘	↘	↘	↘
**CO_2**	↗	↗	⇗	↘	↘	⬊	↘	↘	↘	?

↗ Increasing trend ↘ decreasing trend - No data

↘ ⇘ ⬊ Increasing magnitude

References.

1. Palike, H. *et al.* A Cenozoic record of the equatorial Pacific carbonate compensation depth. *Nature* **488**, 609-+ (2012).
2. Beerling, D. & Royer, D. Convergent Cenozoic CO2 history. *Nat. Geosci.* **4**, 418–420 (2011).
3. DeConto, R. *et al.* Thresholds for Cenozoic bipolar glaciation. *Nature* **455**, 652-U52 (2008).
4. Miller, K., Wright, J. & Fairbanks, R. Unlocking the ice-house Oligocene-Miocene oxygen isotopes, eustasy, and margin erosion. *J. Geophys. Res.-Solid Earth Planets* **96**, 6829–6848 (1991).
5. Boulila, S. *et al.* On the origin of Cenozoic and Mesozoic 'third-order' eustatic sequences. *Earth-Sci. Rev.* **109**, 94–112 (2011).
6. Paxman, G. *et al.* Reconstructions of Antarctic topography since the Eocene-Oligocene boundary. *Palaeoclimatol. Palaeoecol. Palaeogeogr.* **535**, (2019).
7. Pollard, D. & DeConto, R. Continuous simulations over the last 40 million years with a coupled Antarctic ice sheet-sediment model. *Palaeoclimatol. Palaeoecol. Palaeogeogr.* **537**, (2020).
8. Rohling, E. *et al.* Sea level and deep-sea temperature reconstructions suggest quasi-stable states and critical transitions over the past 40 million years. *Sci. Adv.* **7**, (2021).
9. Houben, A., van Mourik, C., Montanari, A., Coccioni, R. & Brinkhuis, H. The Eocene-Oligocene transition: Changes in sea level, temperature or both? *Palaeoclimatol. Palaeoecol. Palaeogeogr.* **335**, 75–83 (2012).
10. Miller, K. *et al.* Cenozoic sea-level and cryospheric evolution from deep-sea geochemical and continental margin records. *Sci. Adv.* **6**, (2020).
11. Smith, Y. *et al.* Icebergs in the Nordic Seas Throughout the Late Pliocene. *Paleoceanogr. Paleoclimatology* **33**, 318–335 (2018).
12. Bailey, I. *et al.* An alternative suggestion for the Pliocene onset of major northern hemisphere glaciation based on the geochemical provenance of North Atlantic Ocean ice-rafted debris. *Quat. Sci. Rev.* **75**, 181–194 (2013).
13. Naafs, B. D. A., Hefter, J. & Stein, R. Millennial-scale ice rafting events and Hudson Strait Heinrich(-like) Events during the late Pliocene and Pleistocene: a review. *Quat. Sci. Rev.* **80**, 1–28 (2013).

14. Hodell, D. A. & Channell, J. E. T. Mode transitions in Northern Hemisphere glaciation: co-evolution of millennial and orbital variability in Quaternary climate. *Clim. Past* **12**, 1805–1828 (2016).
15. Rousseau, D., Bagniewski, W. & Ghil, M. Abrupt climate changes and the astronomical theory: are they related? *Clim. Past* **18**, 249–271 (2022).
16. van de Wal, R. S. W., de Boer, B., Lourens, L. J., Koehler, P. & Bintanja, R. Reconstruction of a continuous high-resolution CO2 record over the past 20 million years. *Clim. Past* **7**, 1459–1469 (2011).
17. Batchelor, C. L. *et al.* The configuration of Northern Hemisphere ice sheets through the Quaternary. *Nat. Commun.* **10**, (2019).
18. Jakob, K. A. *et al.* A new sea-level record for the Neogene/Quaternary boundary reveals transition to a more stable East Antarctic Ice Sheet. *Proc. Natl. Acad. Sci. U. S. A.* **117**, 30980–30987 (2020).
19. Muttoni, G. *et al.* Onset of major Pleistocene glaciations in the Alps. *Geology* **31**, 989–992 (2003).
20. Knudsen, M. F. *et al.* New cosmogenic nuclide burial-dating model indicates onset of major glaciations in the Alps during Middle Pleistocene Transition. *Earth Planet. Sci. Lett.* **549**, (2020).
21. Berends, C. J., de Boer, B. & van de Wal, R. S. W. Reconstructing the evolution of ice sheets, sea level, and atmospheric CO2 during the past 3.6 million years. *Clim. Past* **17**, 361–377 (2021).
22. Seki, O. *et al.* Alkenone and boron-based Pliocene pCO(2) records. *Earth Planet. Sci. Lett.* **292**, 201–211 (2010).
23. Broecker, W. S. & van Donk, J. Insolation changes, ice volumes, and 0-18 record in deep-sea cores. *Rev. Geophys. Space Phys.* **8**, 169–198 (1970).
24. Barker, S. *et al.* 800,000 Years of Abrupt Climate Variability. *Science* **334**, 347–351 (2011).
25. McManus, J. F., Oppo, D. W. & Cullen, J. L. A 0,5-million-year record of millennial-scale climate variability in the North Atlantic. *Science* **283**, 971–975 (1999).
26. Heinrich, H. Origin and Consequences of Cyclic Ice Rafting in the Northeast Atlantic Ocean during the Past 130,000 years. *Quat. Res.* **29**, 142–152 (1988).
27. Bond, G. *et al.* Evidence for massive discharges of icebergs into the North Atlantic Ocean during the last glacial period. *Nature* **360**, 245–249 (1992).

28. Bond, G. *et al.* Correlations between climate records from North Atlantic sediments and Greenland ice. *Nature* **365**, 143–147 (1993).
29. Obrochta, S. P. *et al.* Climate variability and ice-sheet dynamics during the last three glaciations. *Earth Planet. Sci. Lett.* **406**, 198–212 (2014).
30. Elderfield, H. *et al.* Evolution of Ocean Temperature and Ice Volume Through the Mid-Pleistocene Climate Transition. *Science* **337**, 704–709 (2012).
31. Jouzel, J. *et al.* Orbital and millennial Antarctic climate variability over the past 800,000 years. *Science* **317**, 793–796 (2007).
